# External Beam Radiation Therapy and Enadenotucirev: Inhibition of the DDR and Mechanisms of Radiation-Mediated Virus Increase

**DOI:** 10.3390/cancers12040798

**Published:** 2020-03-26

**Authors:** Tzveta D. Pokrovska, Egon J. Jacobus, Rathi Puliyadi, Remko Prevo, Sally Frost, Arthur Dyer, Richard Baugh, Gonzalo Rodriguez-Berriguete, Kerry Fisher, Giovanna Granata, Katharine Herbert, William K. Taverner, Brian R. Champion, Geoff S. Higgins, Len W. Seymour, Janet Lei-Rossmann

**Affiliations:** 1 Anticancer Viruses and Cancer Vaccines Research Group, Department of Oncology, University of Oxford, Oxford OX3 7DQ, UK; tzveta.pokrovska@oncology.ox.ac.uk (T.D.P.); egon.jacobus@oncology.ox.ac.uk (E.J.J.); sally.frost@oncology.ox.ac.uk (S.F.); arthur.dyer@oncology.ox.ac.uk (A.D.); richard.baugh@oncology.ox.ac.uk (R.B.); kerry.fisher@oncology.ox.ac.uk (K.F.); william.taverner@dtc.ox.ac.uk (W.K.T.); janet.lei@oncology.ox.ac.uk (J.L.-R.); 2 Tumour Radiosensitivity Research Group, Department of Oncology, University of Oxford, Oxford OX3 7DQ, UK; rathi.puliyadi@oncology.ox.ac.uk (R.P.); remko.prevo@oncology.ox.ac.uk (R.P.); gonzalo.rodriguez@oncology.ox.ac.uk (G.R.-B.); giovanna.granata@oncology.ox.ac.uk (G.G.); Katharine.Herbert@glasgow.ac.uk (K.H.); geoffrey.higgins@oncology.ox.ac.uk (G.S.H.); 3 PsiOxus Therapeutics Ltd., Abingdon OX14 3YS, UK; brian.champion@psioxus.com

**Keywords:** oncolytic viruses, radiotherapy, virotherapy, combination therapy, DNA damage repair, ionizing radiation

## Abstract

Ionising radiation causes cell death through the induction of DNA damage, particularly double-stranded DNA (dsDNA) breaks. Evidence suggests that adenoviruses inhibit proteins involved in the DNA damage response (DDR) to prevent recognition of double-stranded viral DNA genomes as cellular dsDNA breaks. We hypothesise that combining adenovirus treatment with radiotherapy has the potential for enhancing tumour-specific cytotoxicity through inhibition of the DDR and augmentation of virus production. We show that EnAd, an Ad3/Ad11p chimeric oncolytic adenovirus currently being trialled in colorectal and other cancers, targets the DDR pathway at a number of junctures. Infection is associated with a decrease in irradiation-induced 53BP1 and Rad51 foci formation, and in total DNA ligase IV levels. We also demonstrate a radiation-associated increase in EnAd production in vitro and in a pilot in vivo experiment. Given the current limitations of in vitro techniques in assessing for synergy between these treatments, we adapted the plaque assay to allow monitoring of viral plaque size and growth and utilised the xCELLigence cell adhesion assay to measure cytotoxicity. Our study provides further evidence on the interaction between adenovirus and radiation in vitro and in vivo and suggests these have at least an additive, and possibly a synergistic, impact on cytotoxicity.

## 1. Introduction

Radiotherapy, alongside chemotherapy, immunotherapy and resection, is one of the major treatment modalities in the management of cancer. Current radiotherapy techniques allow improved precision in the treatment of target cells, however, decreasing normal tissue toxicity without compromise in effective tumour eradication remains an ongoing challenge. Radiotherapy uses ionising radiation (IR)-mediated cell death to trigger the induction of DNA damage, particularly double-stranded DNA breaks [1]. The addition of a radiosensitiser can improve the therapeutic window. However, there has been a dearth of radiosensitisers approved for clinical use, with only cetuximab approved for use with radical head and neck radiotherapy in the last decade [2].

Oncolytic viruses are viruses that replicate in and lyse cancer cells. In contrast to other therapies, oncolytic virotherapy is unique in that the initial dose is amplified, potentially allowing for lower administered doses and therefore reduced off-target toxicity. In some cases, oncolytic viruses can also trigger the release of pro-inflammatory pathogen-associated molecular patterns or danger-associated molecular patterns [3,4,5]. A number of viruses, including adenoviruses, are currently being explored as oncolytic agents in the clinic [6,7]. Oncolytic viruses are also being explored as potential radiosensitisers, particularly those with DNA genomes that spend part of their replication cycle inside the host cell nucleus [8,9]. Nuclear-resident viruses, particularly those that have linear genomes such as adenoviruses, are likely to have mechanisms to inhibit the cellular DNA damage response (DDR) that would otherwise allow the cell to detect and mount an antiviral response against the replicating virus.

The vast majority of the published data on adenovirus interactions with the DDR stem from experiments using Ad5-based constructs, though some information is also available on other serotypes. Adenoviral proteins have been shown to interact with members of the DDR at multiple steps along the pathway, both at the level of DNA damage recognition and mechanisms of repair [10]. In particular, prevention of recognition of viral DNA as double-stranded DNA breaks is thought to be a crucial step in the adenovirus replication cycle [11]. This may prevent the DDR machinery from attempting to “repair” these breaks, leading to concatemerisation of the viral genome [11,12].

Sensing of double-stranded DNA breaks is mediated by the MRE11–Rad50–Nbs1 (MRN) complex, and MRN normally activates the ataxia-telangiectasia mutated (ATM) complex, the key mediator of the cellular response to double-stranded DNA breaks. Components of MRN are known to be targeted both for degradation [11,13,14,15,16] and relocalisation [13,14,15,17,18] by adenoviral proteins, but this is not conserved across all serotypes. Forrester et al. found there was no MRE11 degradation following infection with Ad3 or Ad11 and that MRE11 relocalisation following infection with these viruses was to viral replication centres, not to promyelocytic leukemia tracks as was the case with Ad5, Ad9 and Ad4 [15]. Widespread histone 2AX phosphorylation (γH2AX) can be seen following Ad5 infection [19,20] and Ad5- and Ad12- mediated phosphorylation of H2AX is severely impaired by the ablation of ATM expression and partially reduced following the knockdown of ATR [21].

Repair of double-stranded DNA breaks can occur via classical or alternative non-homologous end-joining (NHEJ) or homologous recombination repair (HR). Adenoviral proteins have been shown to interact with proteins involved in NHEJ [22]. Observed in several serotypes, adenoviruses appear to primarily interact with the classical NHEJ pathway through DNA ligase IV degradation [15,23]. The link between adenoviruses and HR is significantly less well established, though data suggests HR proteins seem to be recruited to Ad5 viral replication centres, reportedly improving viral replication and cytotoxicity in cells with functional HR [24].

Adenovirus serotype 5 has been the prevalent adenovirus used in clinical trials to date. The published data available on radiation-associated changes in viral uptake and production is therefore predominantly on replicating and non-replicating Ad5-based viral vectors. [25,26,27,28,29,30,31,32,33]. Enadenotucirev (EnAd) is an oncolytic adenovirus formed as a chimera of two group B adenoviruses, Ad3 and Ad11p [34]. In contrast to Ad5-based vectors, for which neutralising antibodies are widespread in the general population, the seroprevalence of antibodies against group B adenovirus is low [35,36]. EnAd shows impressive selectivity for replication in human carcinoma cells from different types of cancers, including in a co-culture of cancer and normal cells in vitro [37], and has shown a promising targeting and safety profile in an early clinical trial [38]. EnAd can serve as an efficient vector for cancer-selective expression of immune-modulating biologics [39,40,41,42] and can be delivered via the bloodstream into the tumour following systemic administration to humans [43,44,45]. It is currently being tested in clinical trials against solid tumours in combination with several other treatment modalities [38,43].

The aim of this study was to elucidate the relationship between radiotherapy and EnAd. We found that total EnAd production and infectious particle release increased in vitro in irradiated A549 cells and in vivo in irradiated HCT116 xenografts. Though radiation led to a cell-line dependent increase in surface levels of EnAd entry receptors CD46 and desmoglein-2 (DSG), this did not translate to an increase in virus uptake. Instead, the effect appears to be mediated by an increase in the level of adenovirus early gene E1A in pre-irradiated cells. We also demonstrate that EnAd infection is associated with inhibition of radiation-induced 53BP1 and Rad51 foci formation and a decrease in total DNA ligase IV levels. These findings are consistent with an interaction between EnAd and the DDR pathway at multiple levels.

Overall, we show that external beam radiation and EnAd oncolytic virotherapy have an at least additive effect on tumour cytotoxicity. We propose this is likely due to a combination of EnAd’s interaction with multiple DDR pathways and increased virus production, and therefore death by oncosis, in irradiated cultures. Our results will ultimately complement future results from the currently recruiting Chemoradiation with Enadenotucirev as a Radiosensitiser (CEDAR) trial (ClinicalTrials.gov ID: NCT03916510).

## 2. Results

### 2.1. Irradiation of A549 Increases Total EnAd Production

Multiple previous studies have demonstrated that radiation given 24 h post-infection results in improved Ad5 virus production or transgene expression [26,27,46]. Ad5 interactions with the cellular DDR are mediated through early viral proteins, but we also elected to assess the relationship between these two treatments by irradiating infected cells 24 h post infection by the virus. We hypothesise that this would allow time for approximately one replication cycle, thus enabling us to potentially detect the effect of an interaction between viral proteins produced both early and late in the life cycle with the cellular pathways affected by radiation.

**Figure 1:**
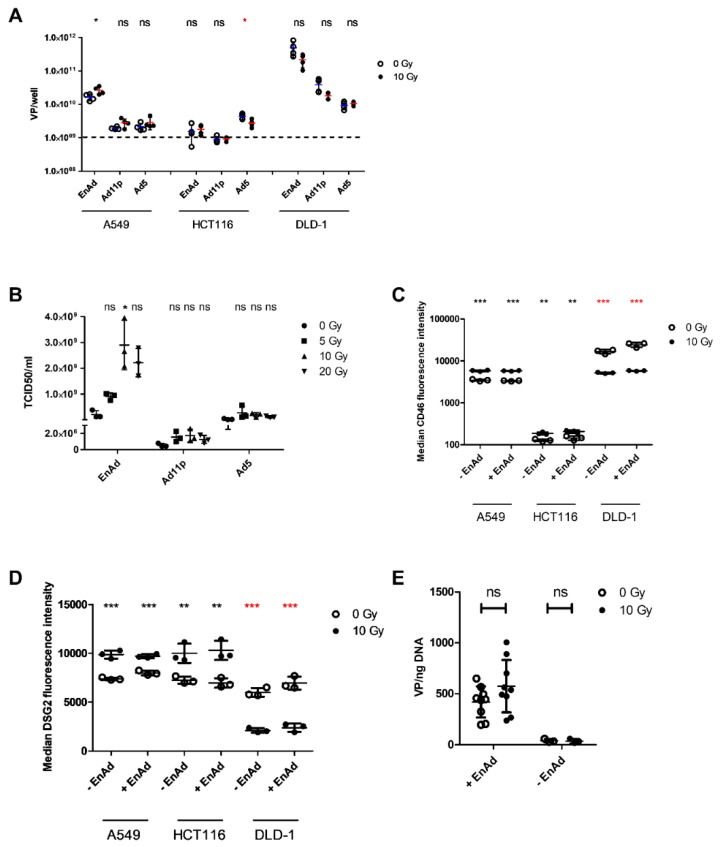
Irradiation increases EnAd genome replication and particle production. (**A**) Viral genomes were quantified at 72 h post-infection (p. i.) in combined supernatant and lysate of A549, HCT116, and DLD-1 cells mock-infected or infected with EnAd-SA-GFP, Ad11p, or Ad5 at an MOI of 0.1. Cells were being irradiated at 10 Gy 24 h post-infection. Genomes were detected using hexon- (EnAd/Ad11p) or fibre-specific (Ad5) primers and probe. Data represents biological replicates ± SD. (**B**) TCID_50_/mL was quantified for supernatant harvested 5 days p. i. from A549 cells mock-infected or infected with EnAd-SA-GFP, Ad11p or Ad5 and irradiated 24 h p. i. at 0, 5, 10 or 20 Gy. Data shows mean ± SD of three experimental repeats, each point representing the mean of three biological replicates within a single experimental repeat. (**C**) CD46 or (**D**) DSG2 median fluorescence levels were measured 24 h after irradiation. A549, HCT116, or DLD-1 cells were infected with EnAd-SA-GFP at an MOI of 0.1 at 24 h before irradiation with 10 Gy. Data represents biological replicates ± SD of a single representative experiment repeated three times. (**E**) A549 cells were irradiated with 10 Gy 18 h prior to seeding in 12-well plates. At 6 h post-seeding, cells were infected with EnAd-SA-GFP at an MOI of 0 or 0.1. At 2 h post-infection, cells were washed twice before extracting and quantifying DNA for viral genomes by qPCR using hexon-specific primers and probes. Data represent biological replicates ± SD from a single experiment representative of three repeats. Significance was evaluated using (**A**,**E**) unpaired two-sided t-test, (**B**) Kruskal–Wallis test with Dunn’s post-hoc testing, (**C**,**D**) two-way ANOVA with Bonferroni post-test. * *p* < 0.05, ** *p* < 0.01, *** *p* < 0.001.

To initially assess whether we could potentiate EnAd particle production and replication using radiation, we measured virus genome copy numbers in irradiated versus non-irradiated A549, DLD-1 and HCT116 cells using qPCR. These cell lines were selected to evaluate radiation’s effect on virus at varying levels of radiosensitivity, A549 and DLD-1 cells being radioresistant, while HCT116 cells are radiosensitive [47,48,49]. Cells were mock-infected or infected at an MOI of 0.1 with EnAd-SA-GFP, Ad11p or Ad5. At 24 h post-infection, plates were mock-irradiated or irradiated at 10 Gy. At 72 h post-infection, we observed an increase in viral genome copy number in EnAd-infected, irradiated A549 cells versus infected, non-irradiated cells (Figure 1A), indicative of an increase in genome production rather than an increase in genome release alone. These findings were not seen for other virus serotypes or cell lines. In fact, there was a significant decrease in irradiated, Ad5-infected HCT116 cells.

### 2.2. Irradiation of A549 Cells Increases EnAd, but not Ad11p or Ad5 Infectious Particle Release

The results discussed above are consistent with an increase in total virus particle production, however, do not indicate whether radiation has an impact on virus infectivity. A549 cells were mock-infected or infected with EnAd-SA-GFP, Ad11p or Ad5 at an MOI of 0.1. At 24 h post-infection, cells were mock-irradiated or irradiated at 5, 10 or 20 Gy. TCID50 was assessed 5 days post-infection. A trend for higher particle concentration was seen in supernatant from irradiated versus non-irradiated cells up until 10 Gy, however post-hoc testing revealed the increase was only significant in EnAd-SA-GFP at 10 Gy (Figure 1B). For all viruses, TCID_50_/mL appeared to be lower in supernatant from cells irradiated at 20 Gy than 10 Gy, supporting the idea that particle production may plateau at higher radiation doses.

### 2.3. Radiation Leads to a Cell Line-Dependent Increase in Surface CD46 and DSG2 Levels

We examined whether the observed increase in viral replication could be a result of increased expression of the group B adenovirus receptors CD46 and desmoglein-2 (DSG2) in irradiated cells. Cells were mock-infected or infected with EnAd-SA-GFP at an MOI of 0.1 PFU/cell. At 24 h post-infection, cells were mock-irradiated or irradiated at 10 Gy. Supernatant and cells were analysed at 48 h p. i. by flow cytometry. Compared to non-irradiated controls, there was a significant increase of CD46 expression levels in irradiated A549 and HCT116 cells (Figure 1C), as assessed by evaluation of median fluorescence intensity, as well as an increase in the percentage of CD46-positive cells (Figures S1A and S7). The presence of EnAd did not change median CD46 expression compared to mock-infected controls. Conversely, CD46 expression decreased in irradiated DLD-1 cells compared to non-irradiated cells. The results for DSG2 median expression levels were similar to those for CD46 (Figure 1D). However, there was a significant decrease in the percentage of DSG-positive cells in irradiated, versus non-irradiated, A549 cells, and no significant change in HCT116 cells (Figure S1B). Additionally, the presence of EnAd increased CD46 median expression levels in DLD-1 cells, but not those of DSG2. These results suggested enhanced binding of virus to target cells may contribute to increased particle production at low MOIs.

### 2.4. EnAd Uptake Is not Affected by Irradiation

The increase in median DSG2 and CD46 expression in A549 cells may be one possible explanation for the observed increase in virus production at low MOIs. Hypothetically, an increase in the number or density of EnAd receptors will increase the probability of virus binding and uptake, leading to a downstream increase in virus production during a multi-step infection. Figure 1C,D demonstrate an increase in median CD46 and DSG2 expression 24 h post irradiation of A549 cells. We therefore used qPCR to quantify EnAd uptake in cells infected 24 h post 10 Gy of radiation. We did not observe any differences between irradiated and non-irradiated cells infected with EnAd-SA-GFP at MOI 0.1 or mock-infected (Figure 1E). These results suggest the increase in virus production seen in Figure 1A is not due to enhanced virus uptake during a multi-step infection and are consistent with receptor level not being a limiting step in virus propagation at this MOI.

### 2.5. EnAd E1A Expression Increases in Pre-Irradiated Cells

Our finding that EnAd uptake does not increase with radiation despite greater receptor expression supports the hypothesis that increased virus production is due to a post-entry, intracellular process. E1A, the first adenovirus gene to be expressed after entry, is an early adenoviral transcriptional unit encoding two principal proteins which play roles in activating transcription and stimulating the cell to enter S phase [50]. To determine whether radiation changes EnAd E1A expression levels or kinetics, we evaluated E1A protein expression in A549 and HCT116 cells infected with EnAd-E1A-FLAG, which expresses E1A C-terminally tagged with the FLAG-tag octapeptide to allow detection using commercial antibodies. Depending on cell line, E1A expression can be detected from approximately eight hours post-infection (Figure S4). We have demonstrated above that irradiation of A549 cells increases virus production at later time points. To establish whether any increase seen in E1A is due to a direct effect of radiation, as opposed to higher levels of virus, we altered the experimental design to carry out infection 24 h post-irradiation. This allowed harvesting of cells prior to viral replication, thus accurate assessment of E1A levels in a single-step infection.

**Figure 2:**
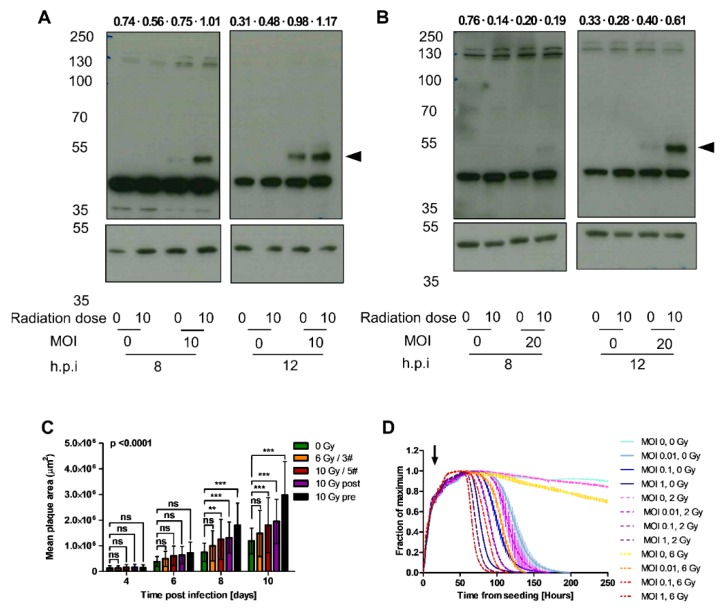
Irradiation increases EnAd particle production and cytotoxicity. (**A**) A549 or (**B**) HCT116 cells were irradiated at 10 Gy and seeded after eight hours. Cells were infected with EnAd E1a-FLAGtag 24 h post-irradiation at an MOI of 0 or 10 (**A**) or an MOI 0 of 20 (**B**). At eight hours and twelve hours post-infection, 70 µg of cell lysates were harvested and probed using an anti-FLAG antibody or β-actin (loading control). Arrows indicate bands of interest. Density for each band is given above the corresponding lane as a β-actin-corrected value. (**C**) A549 cells were pre-irradiated at 10 Gy or 0 Gy at 18 h prior to seeding into 6-well plates at 1 × 10^6^ cells/well. At 6 h post-seeding, cells were infected with EnAd-SA-GFP virus with 0.0001 VP/cell for three hours before overlaying with agarose. At 24 h p. i., cells were either mock-irradiated (for pre-irradiated wells) or irradiated at 10 Gy (“10 Gy post”) or 2 Gy (“10 Gy/5#” and “6 Gy/3#”). Samples marked “6 Gy/3#” were irradiated with a further 2 Gy at 3 and 5 days p. i. Samples marked “10 Gy/5#” were irradiated with 2 Gy at 3, 5, 7 and 9 days p. i. All plates were imaged and analysed using a Celigo imaging cytometer at 4, 6, 8 and 10 days p. i. Data represent biological quadruplicates ± SD from a single experiment representative of two repeats. (**D**) A549 cells were irradiated at 2 or 6 Gy or mock-irradiated at 8 h prior to seeding at 2 × 10^4^ cells/well in E-plates. At 24 h post-irradiation, cells were mock-infected or infected with EnAd-SA-GFP at an MOI of 0.01, 0.1 or 1 (arrow). All conditions were plated in quadruplicate and electrical impendence measured as a model of cell death; outer wells were excluded from analysis. Data represents mean values of biological triplicates ± SD for each sample group compared to the maximum cell index. Significance was evaluated using two-way ANOVA with Bonferroni post-test. ** *p* < 0.01, *** *p* < 0.001.

A strong induction of E1A expression was observed in cells irradiated at 10 Gy 24 h prior to infection compared to non-irradiated cells (Figure 2A,B). This effect was seen in both A549 (Figure 2A) and HCT116 cells (Figure 2B), at both 8 and 12 h p. i. This result suggests that radiation may improve or accelerate virus replication in irradiated cells through an E1A-dependent mechanism.

Interestingly, when the same experiment was conducted with Ad5 including harvesting at 6, 8, 12 and 24 h p. i., an increase was seen in E1A expression in irradiated versus non-irradiated HCT116 cells at 8 and 12 h p. i. (Figure S1D), but not in A549 cells (Figure S1C). Figure 1A demonstrated decreased Ad5 levels in irradiated versus non-irradiated HCT116 cells, which suggests that the mechanisms driving changes in virus production in irradiated cells may differ between adenovirus serotypes. This would plausibly be in keeping with the variable interactions observed between different serotypes and the DDR.

### 2.6. Irradiation Increases Mean Plaque Area

To determine whether the increase in E1A expression and virus genome replication has an impact on viral spread, we examined the formation of viral plaques in A549 cultures irradiated with different radiation regimes. Comparison was made between the effects of radiation delivered pre- or post-infection and in single versus fractionated regimes, to establish the regime which resulted in maximal viral spread through a cell monolayer. Cells were mock-irradiated or treated with a single 10 Gy dose at 24 h pre-infection (“10 Gy pre”), 24 h p. i. (“10 Gy post) or in fractionated doses starting 24 h p. i. (“6 Gy/3#” or “10 Gy/5#”). Cells were mock-infected or infected with EnAd-SA-GFP at 1 × 10^−4^ VP/cell, a dilution optimised to allow tracking of individual plaque growth throughout the experimental time course, and overlaid with agarose. Plates were imaged at 4, 6, 8, and 10 days p. i. on a Celigo imaging cytometer. A dose of 10 Gy of radiation significantly increased mean plaque area, regardless of fractionation, at 8 and 10 days post-infection, compared to mock-irradiated cells (Figure 2C). The largest plaques were seen in cells irradiated with 10 Gy at 24 h pre-infection. These results are consistent with previous experiments and suggestive of maximal augmentation of EnAd plaque growth in vitro when radiation is given in a single high dose 24 h prior to infection.

### 2.7. Irradiation and Infection Act Together to Improve Target Cell Cytotoxicity

Ultimately, a combination of virotherapy and radiotherapy may provide more effective cancer treatment by increasing cancer cell toxicity. To assess this, xCELLigence was utilised to monitor cell adhesion of A549 cells as a proxy for measurement of mono- or dual-therapy-induced cytotoxicity. Cells were irradiated at 2 Gy or 6 Gy, or mock-irradiated 24 h prior to mock infection or infection with EnAd at MOI of 0.01, 0.1 or 1. Published data is suggestive of an enhanced virus response to radiation at lower MOI. A range of virus doses was therefore utilised to establish whether any effect seen in combination treatment was diminished at higher virus doses [26,46]. We observed accelerated cell death in infected, irradiated cells at all MOI, compared to infected or irradiated cells alone (Figure 2D). Bliss independence analysis of the cell index data at each time point revealed that EnAd and radiation are synergistic at all MOIs and radiation doses tested, and co-administration of one does not negatively impact the cytotoxic effect of the other [51]. In fact, 6 Gy of radiation led to cytotoxicity equivalent to levels seen in non-irradiated cells infected with 10 times fewer PFU/cell. Furthermore, over the time frame of this experiment, 2 Gy of radiation caused minimal cell death, comparable to non-irradiated cells. The addition of virus, however, lead to accelerated cytotoxicity in A549 cells irradiated with 2 Gy versus mock irradiation. The finding of augmented virus-induced cytotoxicity at subtherapeutic radiation doses would be more consistent with a synergistic effect on cell death.

### 2.8. Virus Infection Reduces the Number of 53BP1 and Rad51 Foci in Irradiated Cells

The improved cytotoxicity seen in infected, irradiated tumour cells may be due to increased virus production, and therefore virus-mediated oncosis, in irradiated cells. It may also be driven by an increase in radiation-induced cell death secondary to virus inhibition of DNA damage repair. To understand the effect of virus infection on the DDR in both irradiated and non-irradiated individual cells, we explored the formation of DNA damage foci in infected cells, using GFP-expressing EnAd to exclude “noise” from non-infected cells. 53BP1 foci are indicative of double-stranded break repair, particularly promoting NHEJ [52], while Rad51 foci are associated with HR [53], and γH2AX foci denote double-stranded breaks [54]. A549 cells were mock-infected or infected at 500 VP/cell with EnAd-CMV-GFP or Ad5-CMV-GFP, where GFP expression is driven by the constitutive cytomegalovirus immediate-early promoter (CMV). Infection level was optimised to allow minimal cytotoxicity whilst maximising percentage of GFP-expressing cells over the experimental time-course. Cells were left non-irradiated or irradiated 24 h p. i. and analysed at 0, 2, 6, or 24 h post-irradiation for 53BP1, Rad51 or γH2AX foci formation.

**Figure 3:**
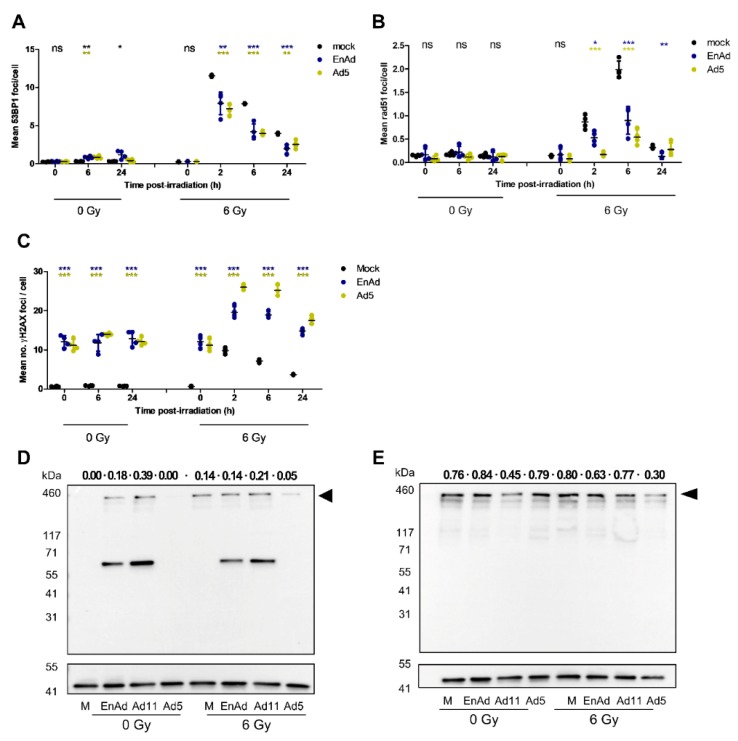
EnAd modulates the DNA damage response in infected cells. (**A–C**) A549 cells were mock-infected or infected with EnAd-CMV-GFP or Ad5-CMV-GFP at 500 VP/cell. At 24 h p. i., cells were either irradiated at 6 Gy or left non-irradiated. Cells were fixed at different time points following irradiation and stained for DAPI and 53BP1 (**A**), Rad51 (**B**) or γH2AX (**C**). Data was acquired and analysed using IN Cell software. Data represent the mean of four biological replicates ± SD of a single experiment representative of three experiments. Significance was evaluated for each time point and virus using two-sided t-test compared to mock-infected control. * *p* < 0.05, ** *p* < 0.01, *** *p* < 0.001. (**D**,**E**) A549 cells were mock-infected or infected with EnAd-SA-GFP, Ad11p or Ad5 at an MOI of 100. Cells were irradiated 22 h p. i. and harvested 24 h p. i. Cell lysates (20 µg/lane) were separated by SDS-PAGE and probed using an antibody against Ser1778-phosphorylated 53BP1 (**D**), total 53BP1 (**E**) or β-actin. m, mock; EA, EnAd; 11, Ad11p; 5, Ad5. Density for each band is given above the corresponding lane as a β-actin-corrected value.

Our results show that radiation-induced induction of 53BP1 foci formation was inhibited by Ad5 and EnAd infection (Figure 3A and Figure S2A), with increased 53BP1 foci formation also observed in infected, non-irradiated cells. An analysis of Rad51 foci formation in irradiated cells also showed a decreased number of foci/cell in adenovirus-infected versus mock-infected wells, consistent with inhibition of the HR pathway (Figure 3B and Figure S2B). There is a long-established impact of adenovirus infection on pan-nuclear γH2AX foci formation [19,20], γH2AX was therefore used as a positive control to confirm that foci detection was not affected by GFP expression. Figure 3C demonstrates that EnAd and Ad5-infected cells contained a significantly greater number of mean γH2AX foci/cell, both in the presence and absence of radiation. This finding supports accurate detection of foci formation in GFP-expressing cells. These results show that adenovirus infection inhibits 53BP1 and Rad51 foci formation, and therefore both NHEJ and HR repair pathways.

### 2.9. EnAd Triggers 53BP1 Phosphorylation in Infected Cells

53BP1 phosphorylation has been shown to be inhibited by E4ORF4, part of the adenovirus E4 early transcriptional unit [55]. Ad5 and Ad11p both encode for functional E4ORF4, while E4ORF4 is truncated in EnAd. To explore whether 53BP1 phosphorylation is inhibited in cells infected with EnAd, A549 cells were infected with 100 PFU/cell of EnAd-SA-GFP, Ad11p, or Ad5, to enable study of potential effects after a single round of infection. At 22 h post-infection, cells were irradiated or mock-irradiated. At 24 h post-infection, lysates were probed for phosphoSer1778-53BP1 and total 53BP1 expression. Irradiation was timed two hours prior to harvesting to coincide with the expected peak of p53BP1 foci formation. Phosphorylation of 53BP1 on Ser1778 was observed in cells infected with EnAd or Ad11p, but not Ad5, regardless of target cell radiation (Figure 3D,E and Figure S5). As expected based on previous literature, 53BP1 phosphorylation increased after irradiation of mock-infected cells. In addition to the expected 53BP1 band at around 450 kDa, we also observed a band of around 60 kDa in group B adenovirus-infected samples (EnAd and Ad11p). This band could indicate a cleavage or degradation product of 53BP1, potentially a result of incomplete degradation due to differences in E4 between group B and C adenoviruses, but not due to a lack of functional E4ORF4. This phosphorylation and cleavage pattern was also observed in HCT116 cells when this experiment was repeated with mock, EnAd or Ad5 infection (Figures S3 and S8).

### 2.10. EnAd Causes a Decrease in DNA Ligase IV Levels that Is Partially Dependent on Proteosomal Degradation

**Figure 4:**
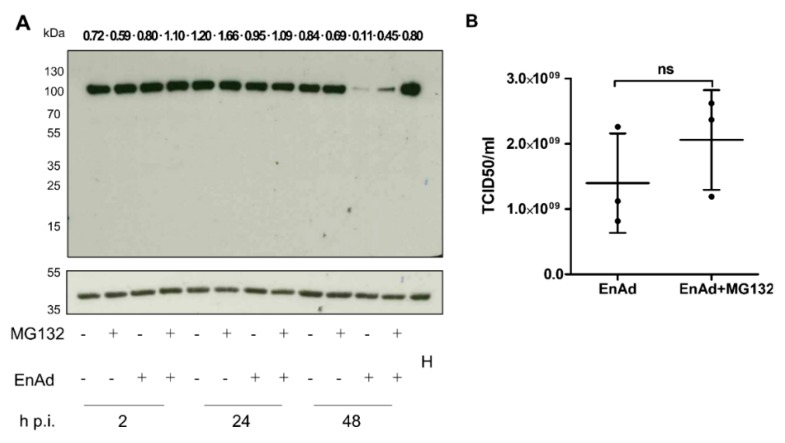
Enadenovirus infection is associated with a partially proteasomally mediated decrease in DNA ligase IV levels. A549 cells were infected with EnAd-SA-GFP at 200 VP/cell (approximately an MOI of 11) or mock-infected. At 2 h post-infection, cells were treated with 10 µM MG132 or DMSO control. (**A**) Cell lysates were harvested either immediately after treatment, 24 h p. i., or 48 h p. i., separated by SDS-PAGE and probed with antibodies against DNA ligase IV or β-actin. Lanes contain 30 µg of protein. Data is representative of three repeats. Density for each band is given above the corresponding lane as a β-actin-corrected value. H, HeLa cell lysate used as a positive control for DNA ligase IV detection. (**B**) Culture supernatants were harvested 48 h p. i. to determine TCID_50_/mL. Data represents mean results from individual experiments performed in triplicate ± SD. Significance was evaluated using a two-sided *t*-test.

DNA ligase IV is another component of the DDR machinery. Published data has shown that the decrease in DNA ligase IV following infection is proteasomally mediated in Ad5-infected cells [23]. DNA ligase IV is thought to be targeted by a E3 ubiquitin ligase complex of E4ORF3, E4ORF6, and E1B-55K. We therefore investigated whether this was also the case for EnAd. A549 cells were infected with EnAd-SA-GFP at 200 VP/cell or mock-infected. At 2 h p. i., cells were treated with the proteasome inhibitor MG132 or a DMSO vehicle control. A decrease in DNA ligase IV is seen at 48 h p. i. in EnAd-infected cell lysates. This decrease is partially abrogated by treatment with MG132 (Figure 4A and Figure S6). The addition of the proteasome inhibitor MG132 does not lead to a significant change in infectious particle production, as measured by TCID_50_ (Figure 4B). This indicates that EnAd also mediates DNA ligase IV degradation through the proteasome, though it is still unclear whether this involves adenovirus E3 ubiquitin ligase complexes as with Ad5.

### 2.11. Irradiation of Mouse Tumours Increases Viral Vector Transgene Expression

We evaluated the impact of radiation on EnAd production in a pilot in vivo experiment to establish if this was consistent with findings in vitro. As adenoviruses are unable to replicate in murine cells [37,56], we used an immunodeficient SCID mouse model to allow for human HCT116 xenografts. The HCT116 cell line was selected to assess the in vivo interaction between EnAd and radiation in colorectal-derived cells, thus aiming to complement the current CEDAR trial recruiting patients with locally advanced rectal cancer. The Phase 1 Mechanism of Action Trial for EnAd found that intravenous injection of EnAd resulted in successful delivery of virus to a range of tumour types, including colorectal [43].

**Figure 5:**
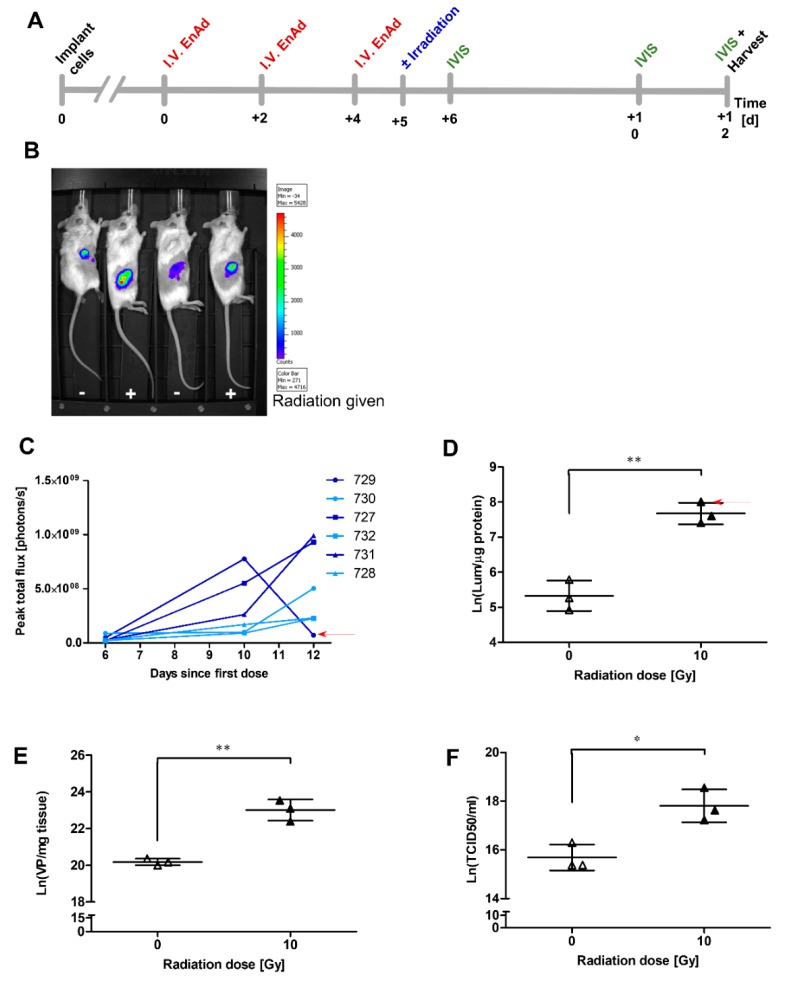
HCT116 xenograft model of tumour irradiation and virus replication. HCT116 cells (2 × 10^6^ cells/mouse) were implanted subcutaneously into six SCID mice. When tumours reached a volume of 50 mm^3^, all mice were given three intravenous injections of 0.5 × 10^10^ virus particles EnAd-SA-FireflyLuciferase (EnAd-SA-Fluc) 48 h apart. One day after the third dose, half of the animals were randomised to receive 10 Gy of radiation to the tumour. Over the next 7 days, animal weight and tumour volume were monitored. Luciferase expression was monitored using an In Vivo Imaging System (IVIS). Mice were sacrificed and tumours were extracted at 12 days after the first virus dose for downstream analysis. (**A**) Experimental timeline. (**B**) Representative IVIS image demonstrating luminescence seen in irradiated (+) and non-irradiated (−) mice. Colour scale represents increasing total flux (photons/second), with blue representing a minimum of 271 p/s and red representing a maximum of 4716 p/s. (**C**) Peak total flux for all pairs of animals. Tumours were imaged at 6, 10 and 12 days after the first dose of EnAd-SA-Fluc. (**D**) Luciferase expression was measured in fresh tumour homogenate harvested immediately after sacrifice using a PolarStar Omega plate reader. (**E**) DNA was extracted from tumour homogenate to measure viral genomes by qPCR using hexon-specific primers and probe. (**F**) Viral TCID_50_/mL from tumour homogenate was evaluated using A549 cells. Data represents single animals. Significance was evaluated using logarithmic transformation to normalise data thus allowing the use of an unpaired t-test. Figures show mean ± SD; * *p* < 0.05, ** *p* < 0.01.

HCT116 cells (2 × 10^6^/mouse) were injected subcutaneously into six SCID mice. When the resulting tumours reached a volume of 50 mm^3^, all mice were given three injections i.v. of 0.5 × 10^10^ VP EnAd-SA-Firefly Luciferase (EnAd-SA-Fluc) intravenously 48 h apart, mirroring the virus dosing schedule and route of administration used in the Phase I clinical trial [38,43] and to minimise the risk of tumour ulceration that may occur following injection and irradiation of the same area using intratumoural administration. One day following the final virus dose, mice were randomised to receive either 10 Gy of irradiation to the tumour or to remain non-irradiated, in keeping with the dose and time point found to have a significant impact on virus production in vitro. At 6, 10 and 12 days after the first dose of EnAd-SA-Fluc (corresponding to 1, 5, or 7 days post-irradiation), mice received a subcutaneous dose of luciferin and underwent imaging using the IVIS to assess intratumoural luciferase expression. Tumours were harvested at a pre-determined time point 12 days after the first dose of virus (corresponding to 7 days post-irradiation). Weight and tumour volume were regularly monitored throughout the experiment. Figure 5A summarises the experimental timeline.

To measure the delivery, distribution, and replication activity of EnAd-SA-Fluc in the animals, luciferase activity was measured by the In Vivo Imaging System (IVIS). Following subcutaneous injection of luciferin, mice were scanned every minute. Scanning was discontinued when peak luminescence or the maximum time allowed for maintaining animals under general anaesthesia was reached, whichever was soonest. Tumour luminescence was determined by using the Living Image software package to identify a “region of interest” over the tumour and a region representing “background fluorescence” over the head to calculate total flux in the area of the tumour (Figure 5B). Figure 5C demonstrates that peak total flux is elevated at later time points in two of three irradiated mice compared to non-irradiated animals, indicating accumulation and replication of the virus at the site of the tumour. The third irradiated mouse (729, arrow), shows an end-point peak flux level approximating baseline levels. This result may be due to decreased virus replication at the site of the tumour, or to failure of luciferin delivery, which can occur if there is leakage of the drug during subcutaneous injection. Figure 5D shows data from a luciferase assay carried out on fresh tissue from these animals, harvested immediately post-sacrifice. The highest luminesce value (arrow) corresponds to mouse 729, the animal which appears to have the lowest levels of luminescence at day 12 in Figure 5C. This would be consistent with decreased peak flux secondary to failure of luciferin delivery, as opposed to decreased virus replication. To quantify the viral load in the tumour, we also measured the number of viral genome copies per mg of resected tumour tissue after sacrifice (Figure 5E). The infective potential of the EnAd-SA-Fluc particles obtained from fresh frozen tissue was assessed by evaluating TCID_50_/mL of homogenate (Figure 5F). The data obtained from fresh tissue samples did not appear to be normally distributed, a finding frequently seen in this type of experiment. Normalisation was therefore carried out by taking natural logarithms, allowing statistical assessment through the use of unpaired t-test.

In fresh tumour tissues, there was an increase in luciferase production, VP production and infectious particle production in irradiated versus non-irradiated tumours. These results correlate well with results seen in A549 cells in vitro. Despite no significant increase in EnAd virus particle production in irradiated HCT116 cells compared to mock-irradiated cells in the qPCR experiment described in Figure 1A, EnAd E1A expression was markedly enhanced in pre-irradiated HCT116 cells (Figure 2B). The in vivo data presented here would be consistent with a radiation-mediated increase in EnAd production in vivo. Hypothetically, the microenvironmental and radiobiological factors present in a 3D environment may amplify the effect of radiation on EnAd production in this cell line compared to a 2D monolayer.

### 2.12. Addition of EnAd to Irradiation Slows Growth of Mouse Tumours

**Figure 6:**
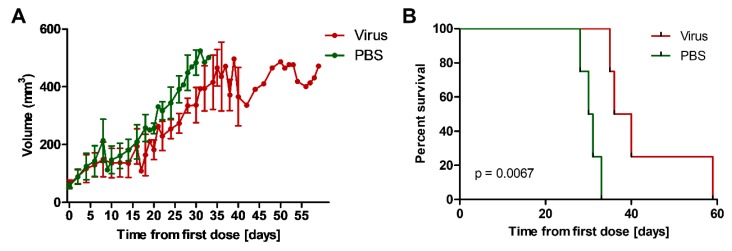
In vivo HCT116 xenograft model shows a significant tumour growth delay. HCT116 cells (2 × 10^6^ cells/mouse) were implanted subcutaneously into eight athymic nude mice. Tumours were monitored until they reached a volume of 50 mm^3^ before randomisation into two groups. Four mice each were given three intravenous injections of either 0.5 × 10^10^ virus particles EnAd-SA-Fluc or 50 μL phosphate buffered saline 48 h apart. One day after the final dose, all animals received 10 Gy of radiation to the tumour. Animal weights and tumour volumes were monitored until a tumour volume of 500 mm^3^ was reached and the mice were sacrificed. (**A**) Tumour volumes and (**B**) survival curves for both groups. Significance was assessed using log-rank test. Graph shows mean of individual mice in each group ± SD.

We have demonstrated that EnAd infection is associated with inhibition of the DDR pathway at several levels. We therefore wished to assess whether the addition of EnAd given prior to radiation, thus allowing for potential radiosensitisation, had an impact on tumour growth in a subcutaneous HCT116 xenograft model [37]. Mice were randomised into two groups once tumour volume reached a pre-determined size of 50 mm^3^. Half received three intravenous doses of 0.5 × 10^10^ virus particles of EnAd-SA-Fluc 48 h apart and half received three intravenous doses of 50 µl PBS 48 h apart. One day after the final dose, all animals received 10 Gy of radiation to the tumour. Animal weight and tumour volume were monitored until a tumour volume of 500 mm^3^ was reached, at which point the animals were sacrificed (Figure 6A). We observed a significant increase in survival time between start of treatment to time of death of irradiated mice in the EnAd- versus PBS-treated group (median 38 versus 30.5 days, respectively) and percent survival using the log-rank test (Figure 6B). These results were consistent with a significant tumour growth delay in mice treated with EnAd, versus mice treated with PBS, when a single 10 Gy dose of radiation was given to the tumour 24 h after the final injection. These results are suggestive of a potential radiosensitising effect of EnAd when this is delivered prior to radiation in vivo.

## 3. Discussion

In this study, we showed that EnAd replication and particle formation increased in irradiated A549 cells in vitro and HCT116 cells in vivo. Despite a radiation-dependent increase in CD46 and DSG2, it was higher transcription of the adenovirus early gene E1A, rather than virus uptake, that seemed to drive the improvement in viral replication. A combination of EnAd and radiation was found to be synergistic in driving cell killing over time. Congruent with these results, irradiation of target cells led to better virus spread and larger plaque formation, eventually leading to improved target cell toxicity. Molecularly, we found that EnAd increased 53BP1 phosphorylation on Ser1778, thus demonstrating interaction with a key protein in the DDR pathway [57]. In vivo pilot experiments show increased EnAd transgene expression with radiation and a delay in tumour growth when EnAd is used as a radiosensitiser. Indeed, the in vivo data indicate an even greater interaction between EnAd and radiation than our in vitro data would suggest, possibly due to the tumour microenvironment in the 3D model resulting in greater radioresistance.

Forrester et al. have previously demonstrated DNA ligase IV degradation following infection with Ad3 and Ad11, both viral serotypes forming the chimeric EnAd [15]. We confirm this finding in EnAd-infected cells and further show the DNA ligase IV degradation to be at least partially proteasomally mediated. This is in keeping with data suggesting that E1b55k/E4ORF6, both early virus proteins, mediate proteasomal degradation of DNA ligase IV in cells infected with adenovirus serotype 5 [23]. Though the interaction between adenoviral proteins and the NHEJ pathway is relatively well established, we are the first to show inhibition of Rad51 foci formation in adenovirus-infected cells. Co-localisation between viral replication centres and HR has previously been demonstrated with Ad5; however, results were consistent with no effect on HR efficiency in the models used [24].

We hypothesise that EnAd-mediated inhibition of the DDR may be secondary to early viral proteins, given that is the primary mechanism in Ad5 and would result in inhibition before exposure of replicating viral genomes to host proteins. Further, the increase seen in viral production following radiation may be partially due to this inhibition being imperfect or incomplete. Thus, sequestration of DDR proteins away from viral replication centres toward cellular dsDNA breaks induced by radiation may allow better viral replication. Indeed, cellular DNA damage induced by chemotherapeutic drugs has been shown to rescue genome replication of Ad5 viruses lacking genes involved in DDR inhibition [16]. We also observed that viral genomes increase with radiation in A549 cells but not HCT116 or DLD-1 cells (Figure 1A). Differences in the response to radiation in these cell lines could stem from the ability of these cells to undergo DNA mismatch repair. A549 cells are microsatellite-stable, while HCT116 and DLD-1 cells are highly microsatellite-unstable [58,59]. Given that uptake does not seem to play a major role in the increase of replication in A549 cells (Figure 1E), we speculate that any potential increases due to E1A production in HCT116 or DLD-1 cells could be negated by an insurmountable increase in the error rate of viral genome replication. However, further studies would be necessary to confirm the impact of microsatellite instability status on adenovirus replication.

A number of interactions between the ATM/ATR pathways and adenovirus proteins have been demonstrated, and the effects on ATR substrates have been shown to differ between adenovirus serotypes [15,21]. Though expression of adenoviral serotype 5 (Ad5), protein E4ORF4 has been linked to decreased phosphorylation of ATM pathway proteins [55], all adenoviral serotypes, including Ad5, have been shown to increase ATM activation in HeLa cells [15]. This is hypothesised to be a consequence of two different mechanisms of ATM activation: a global mechanism which does not influence viral replication but is detectable by standard assays, and a localised, MRN-dependent, antiviral mechanism which is inhibited as part of the viral replication cycle. Thus, though viral replication results in localised inhibition of the MRN-ATM pathway, there is net overall ATM phosphorylation [16].

The protein p53, a central player in the cellular response to DNA damage and stress, appears to also be differentially targeted by different serotypes. Effects of adenovirus infection range from degradation to an apparent increase in p53 levels, though this increase does not result in a corresponding increase in p53 transcriptional activity [15]. Instead, the majority of serotypes demonstrating an increase in p53 levels appear to cause p53 sequestration to viral replication centres [15], and viral E1A and p53 interaction is thought to be involved in major late promoter function [60]. Thus, though p53 protein levels increase following infection with specific adenoviral serotypes, normal p53 function is subverted to aid the virus.

An integral part of assessing the potential for synergy between oncolytic adenovirus therapy and radiotherapy involves investigating the effect of radiation on virus. As discussed above, radiation-induced cell death is primarily due to induction of double-stranded DNA breaks. It would not be unreasonable to query whether dsDNA break induction in virus particles may interfere with their capacity for spread and infection. Statistically, this would be unlikely, simply based on the relative size of the viral genome (approximately 36 kb) in comparison to the human genome (3 × 10^6^ kb). A review of the literature is in fact in keeping with enhanced adenovirus production when infection is combined with radiation treatment. As would be expected, this is not seen consistently for all cases, but appears to be dependent on the cell line, MOI, radiation dose and timing of treatment used.

Using standard UK radiotherapy regimes, patients treated with curative intent will commonly receive radiation in doses approaching 2 Gy per fraction, whereas patients treated with palliative intent can be treated with a single 8 Gy fraction. As our results above show that both a single higher dose and fractionated radiation potentiates virus activity (Figure 1 and Figure 2), we anticipate that improved efficacy of virus activity in patients can therefore be achieved using a range of regimes, both palliative and radical.

The Chemoradiation with Enadenotucirev as a Radiosensitiser (CEDAR, ClinicalTrials.gov: NCT03916510) trial in locally advanced colorectal cancer, led by Maria Hawkins, is currently recruiting. This Phase I study will evaluate the safety of co-administration of EnAd and chemoradiotherapy in patients with locally advanced rectal cancer. The aim of the study is to determine the toxicity profile and maximum tolerated dose of the combination in patients. The planned radiation dosing is 50 Gy over 25 fractions, with 2 Gy per fraction. Our studies therefore correspond well to the dose that is to be used in the CEDAR trial. The combination treatment will be administered prior to surgery and will inform further Phase I efficacy studies, including potential side effects such as GI or haematological toxicity, or cytokine release syndrome.

In addition to colorectal cancer, other cancers, particularly those in which radiotherapy and immunotherapy play key roles, could be good candidates for combination radiotherapy and oncolytic virotherapy. One prime example of this is lung cancer, one of the commonest types of malignancy, which is nonetheless associated with markedly low 5-year survival. Immunotherapy has now become one of the standard treatment options for patients with advanced non-small cell lung cancer. Dyer at al. have provided evidence that EnAd induces an immunogenic cell death [5]; it may therefore provide a useful adjunct alongside this treatment modality. The primary cell line utilised in vitro in this study is A549, derived from a lung carcinoma and selected because of its relative radioresistance. We have shown that in this lung cancer-derived cell line there is a significant increase in EnAd production and enhanced cytotoxicity when cells are exposed to radiation. Moreover, in an orthotopic and subcutaneous A549 mouse model, treatment with EnAd or transgene-expressing EnAd-based vectors led to a significant increase in survival compared to untreated controls [39].

Several studies have shown that EnAd virotherapy is also particularly suitable for systemic administration to target metastases, providing benefit over other oncolytic viruses which require intratumoural administration. Thus, visceral metastases, such as those in the liver which may not otherwise be easily accessible, can be targeted through intravenous administration, as EnAd is stable in whole blood, entering tumour deposits through the basolateral surface [45]. Previous clinical trial data has shown that EnAd administered intravenously over three doses 2 days apart can localise to the primary tumour site and promote infiltration of CD8+ T cells [43].

We are also currently exploring the possibility of “arming” EnAd with different biologics to provide a two-, or in the case of combination with radiotherapy, three-pronged attack on the tumour microenvironment. In this way, armed EnAd viruses can target not only tumour cells [40], but also other tumour-promoting cells, such as fibroblasts [41] and M2-polarised, wound-healing macrophages [42] that form part of a tumour’s support network. Though EnAd does not replicate in or lyse these latter cell types effectively, bi- and tri-specific T cell engagers encoded within the genome of EnAd can be secreted from infected tumour cells. These T cell engagers directly couple target cells to T cells in the immediate vicinity, such as CD8+ T cells that infiltrate the tumour site due to EnAd infection, as observed in a Phase I mechanism of action study. This immune activation may also inhibit some of the immunosuppressive effects of radiotherapy, such as temporary upregulation of TGFβ in the tumour microenvironment [61], suppression of CD8+ T cells and promotion of Tregs [62].

Limitations of our study include the restriction of our findings to EnAd and related group B adenoviruses. Much of the work on the interaction between adenoviruses and radiotherapy is based on Ad5, and this study finds that the interaction between radiotherapy and group B adenoviruses differs and is perhaps even more therapeutically promising than that for group C viruses. Indeed, the results of this study further justify the Phase I CEDAR trial, which is currently recruiting patients.

## 4. Materials and Methods

### 4.1. Mammalian Cell Culture

A549 human lung carcinoma, DLD-1 and HCT116 human colorectal carcinoma cells were cultured in DMEM supplemented with 10% foetal calf serum (complete culture medium) at 37 °C and 5% CO_2_. All cell lines were routinely tested for mycoplasma. A549 was obtained from EACC, DLD-1 from ATCC and HCT116 was a gift from the McKenna Lab (Oxford University).

### 4.2. In Vitro Irradiation

Cells were treated in T-75 tissue culture flasks or Corning Costar plates using a caesium-137 irradiator.

### 4.3. Infection Studies

Unless otherwise stated, infections were performed in culture medium supplemented with 2% foetal calf serum for two hours at 37 °C before changing the medium for complete fresh culture medium. Details of MOI utilised in individual experiments are given below. Briefly—as EnAd causes a high level of cytotoxicity in vitro—in experiments assessing the effect of radiation on virus, cells were infected at MOI 0.1 to allow a longer period for observing the impact of radiation on virus replication. Conversely, infection at high MOI was conducted in experiments assessing the effect of virus on intracellular pathways to increase the number of infected cells and thus maximise the probability of such an effect being detected.

For receptor expression and virus production studies in irradiated cells, cells were seeded into 12-well or 24-well plates, respectively, and mock-infected or infected at an MOI of 0.1 with EnAd-SA-GFP the next day. At 24 h post-infection, plates were mock-irradiated or irradiated at 10 Gy. Cells and supernatant were harvested at 48 h post-infection for receptor expression studies or 72 h post-infection for DNA extraction and virus genome quantification (total virus production).

To quantify particle release, A549 cells were seeded into 96-well plates and the subsequent day mock-infected or infected with EnAd-SA-GFP, Ad11p or Ad5 at an MOI of 0.1. Cells were mock-irradiated or irradiated at 5, 10 or 20 Gy at 24 h p. i. Cell supernatant was harvested 5 days post infection and TCID_50_ (50% Tissue Culture Infective Dose)/mL concentration determined by serial titration on A549 cells seeded in 96-well plates at 10,000 cells/well. The highest dilution to show cytopathic effect after 7 days was used to calculate the TCID**_50_** using the Spearman–Karber method.

For 53BP1 phosphorylation studies, A549 cells were seeded in six-well plates and infected the next day with 100 PFU/cell of EnAd-SA-GFP, Ad11p, or Ad5. Half of the wells were irradiated at 22 h p. i. and all cells lysed at 24 h p. i. for immunoblot analysis, probing for phosphoSer1778-53BP1 and total 53BP1 expression.

For proteome inhibitor studies, A549 cells were seeded into 6-well plates overnight and infected with EnAd-SA-GFP at 200 VP/cell or mock-infected. At 2 h p. i., cells were treated with the proteasome inhibitor MG132 or a DMSO vehicle control. Lysates were harvested either immediately following addition of MG132/DMSO, or at 24 or 48 h p. i. Culture supernatants were harvested to quantify the number of released infectious particles by TCID_50_/mL.

### 4.4. Quantitative PCR

Viral genomes were measured by quantitative PCR using primers and probes specific for the hexon or fibre genes for EnAd/Ad11p or Ad5, respectively. Genomic DNA was extracted from harvested cells using the PureLink Genomic DNA Purification Kit (Life Technologies, Loughborough, UK, #K182001). Extracted DNA genomes were quantified in a 20 μL qPCR reaction consisting of 2× qPCRBIO Probe Mix Hi Rox (PCR Biosystems, London, UK), 400 nM each of forward primer and reverse primer and 200 nM of probe. Cycling conditions were as follows: one cycle at 95°C for 2 min, followed by 40 cycles at 95 °C for 5 s and 60 °C for 20 s. Ct values from known quantities of virus particles were used to calculate a standard curve. EnAd/Ad11p forward primer: 5′ TACATGCACATCGCCGGA 3′, EnAd/Ad11p reverse primer: 5′ CGGGCGAACTGCACCA 3′, EnAd/Ad11p probe: [6FAM] CCGGACTCAGGTACTCCGAAGCATCCT [TAM]. Ad5 forward primer: 5′-TGGCTGTTAAAGGCAGTTTGG-3′, Ad5 reverse primer: 5′-GCACTCCATTTTCGTCAAATCTT-3′ and Ad5 fibre probe: [6FAM] TCCAATATCTGGAACAGTTCAAAGTGCTCATCT [TAM].

### 4.5. Immunoblotting and Immunocytochemistry

Protein expression in infected cells was analysed by immunoblotting. Infected cells were harvested by removing the supernatant from cell cultures and rinsing gently with PBS. Cells were lysed by adding Pierce RIPA buffer supplemented with 1 × protease inhibitor directly to the cell monolayer and incubating on ice for 10 min. Lysates were scraped and transferred into 2 mL Eppendorf tubes, incubated with 0.25 μL Benzonase for 30 min at room temperature and centrifuged for 10 min at 10,000 *g*, 4 °C. Lysate concentrations were measured by the QuantiPro BCA Assay Kit (Sigma Aldrich, Gillingham, UK, QPBCA-1KT). Samples containing 20–70 µg of each protein lysate in 1× Laemmli sample buffer were heated at 95 °C for 15 min. Proteins in the experiment investigating EnAd-E1A-FLAGtag expression were separated on a 10% Mini PROTEAN TGX Precast Protein Gel (Bio-Rad, Watford, UK), all other experiments utilised a 4–20% Mini PROTEAN TGX Precast Protein Gel (Bio-Rad, UK). Transfer was onto a 0.45 μm nitrocellulose membrane when blotting for p53BP1 or 53BP1 and onto 0.2 μm nitrocellulose membrane for all other experiments. The semi-dry transfer method was utilised for experiments investigating E1A expression and the wet blot method for all other experiments. Blots were probed with the following antibodies, as indicated in the relevant figure: rabbit mAb anti-DNA ligase IV (1:500, Cell Signalling Technologies #14649), rabbit polyclonal Ab anti-Phospho-53BP1 (Ser1778) (1:1000, Cell Signalling Technologies #2675), rabbit polyclonal Ab anti-53BP1 (1:1000, Cell Signalling Technologies #4937), rabbit polyclonal Ab anti-Rad51 (1:1000, Santa Cruz Biotechnologies #sc-8349), goat polyclonal Ab anti-adenovirus (1:20,000, Abcam #ab36851), rat monoclonal Ab anti-FLAGtag conjugated to horseradish peroxidase (1:5000, Biolegend #637311), mouse monoclonal Ab anti-β-actin conjugated to horseradish peroxidase (1:400,000, Sigma-Aldrich #A3854). Antibodies used for immunocytochemistry were: mouse anti-53BP1 (1:1000, BD Transduction #612523), rabbit anti-Rad51 (1:1500, Santa Cruz Biotechnologies # sc-8349), rabbit anti- γ-H2AX [pSer139] (1:2000, Novus Biologicals #nb100-2280), goat anti-rabbit IgG conjugated to Alexa Fluor 488 (1:1200, Invitrogen #A11070). Horseradish peroxidase-conjugated goat anti-rabbit IgG (1:3000, Cell Signalling Technologies #7074), horse anti-mouse IgG (1:3000, Cell Signalling Technologies #7076), or mouse anti-goat IgG (1:10,000, Santa Cruz Biotechnologies #sc-2354) were used as secondary antibodies to detect unconjugated primary antibodies. Membranes were incubated with SuperSignal West Dura Extended Duration Substrate (Thermo Fisher, Loughborough, UK, #34075) and bands detected using the Gel Doc system (Bio-rad) or through exposure to X-ray film. Quantification of blots was performed by densitometry using image lab software. Intensity volume of bands was normalized to the relevant loading control (β-actin) band.

### 4.6. xCELLigence Studies

The xCELLigence RTCA DP instrument (Roche) was used for the real-time monitoring of cell growth and cytotoxicity. A549 cells were irradiated or mock-irradiated and eight hours later added to pre-warmed complete medium in 16-well E-Plates (ACEA Biosciences, San Diego, CA, USA) at 20,000 cells per well. A baseline reading was performed prior to addition of cells. At 24 h post-irradiation complete medium with or without EnAd was added at the doses indicated. Impedance measurements for each well were then taken automatically every 15 min and expressed as a cell index (CI). Synergy was evaluated by calculating the Bliss independence factor E_ind_ = E**_a_** + E**_b_** − E**_a_** × E**_b_**, where E**_a_** is the cell index of cultures treated with virus alone and E_b_ is the cell index of cultures treated with radiation alone at a given time point [51]. Deviations of the observed cell index of the combination treatment from the predicted cell index as calculated by Bliss were given as ΔE = E_obs_ − E_ind_. Values of ΔE > 0 indicate a synergistic interaction, while ΔE ≤ 0 indicate an antagonistic interaction.

### 4.7. Assessment of Viral Plaque Growth by the Celigo Image Cytometer

A549 cells were pre-irradiated at 10 Gy or left non-irradiated and 18 h later seeded into 6-well plates at a density of 1 × 10^6^ cells/well. Six hours post-seeding cells were infected with EnAd at 1 × 10^−4^ virus particles/cell or mock-infected; the medium was aspirated 3 h post infection and replaced with 1% agarose + DMEM + 2% FBS. At the indicated time points post infection, cells were irradiated or mock-irradiated. Images were obtained in both brightfield and fluorescent channels using the Celigo image cytometer (Nexcelom Biosciences, Manchester, UK) and analysis carried out by the Celigo software using the Colony program. This program measures the surface area of separate GFP positive “targets” in the fluorescent channel.

### 4.8. Luciferase Reporter Assay

Fresh frozen tissue from mouse tumour samples was homogenised in PBS supplemented with 1% pen/strep (10 μL/mg tissue) and 1:200 Halt protease and phosphatase inhibitor, and 50 μL of this diluted into PBS 1:2. Cells were lysed in reporter lysis buffer (Promega) using one freeze-thaw cycle. Supernatant was plated in triplicate in white 96-well plates and luciferase activity was measured using the Promega luciferase assay system (Promega, Southhampton, UK, #E1500) following the manufacturer’s instructions using a POLARstar Omega plate reader spectrophotometer (BMG Labtech).

### 4.9. Flow Cytometry

Cells were seeded into 12-well plates and allowed to adhere overnight prior to mock-infection or infection with EnAd-SA-GFP at MOI of 0.1. At 24 h p. i cells were irradiated at 10 Gy or mock-irradiated. Supernatant and cells were harvested at 48 h p. i. and stained with LIVE/DEAD Fixable Near IR stain (#L34975, Invitrogen) prior to fixing in 2% formalin (Sigma-Aldrich). Staining with PE-conjugated CD46 at a dilution of 1:200 (#352401, BioLegend), Desmoglein 2 antibody at a dilution of 1:20 (#12-9159-42, Invitrogen), or appropriate isotype control (#400114, BioLegend for CD46 and #12-4732-81, Invitrogen for DSG2), was performed in MACS buffer (0.5% BSA, 2 mM EDTA in PBS). Data was acquired on an Attune NxT acoustic focusing cytometer (Life Technologies). Analysis was conducted using FlowJo analysis software (Becton & Dickinson).

### 4.10. In Vivo Studies

Animal experiments were carried out in accordance with the UK Home Office guidelines under the Animals (Scientific Procedures) Act 1986 under PPL Number 30/3391 (approved 16-March-2016) and the UKCCCR Guidelines for the Welfare of Animals and were approved by the University of Oxford Animal Welfare and Ethical Review Body (AWERB). All animals were held in individually ventilated cages in specific pathogen-free barrier units and allowed to acclimatise for 1 week prior to any procedures being carried out.

For assessing the effect of radiation on virus production, HCT116 (2 × 10^6^ cells, n = 6), were inoculated subcutaneously on the right flank of SCID mice. Once a palpable tumour was apparent, tumour growth was monitored until a volume of 50 mm^3^ (V = L × W × D × pi/6) was reached, at which point all animals were treated with three doses of 0.5 × 10^10^ particles of EnAd-SA-FLuc virus in 50 µL PBS, given i.v. 48 h apart. Administration of virus was performed in a procedure chamber equipped with a HEPA filter. Animals were randomly assigned 24 h post final dose to receive 10 Gy of radiation (n = 3), or no radiation (n = 3), delivered using a Gulmay 320 kV irradiator. IVIS imaging was performed after treatment (3 imaging sessions) to monitor virus-mediated expression of firefly luciferase. Before imaging, 150 mg/kg D-luciferin dissolved in 100 µL sterile PBS was administered subcutaneously. Animals were anesthetised for the imaging procedure with isoflurane. Tumours were harvested 8 days after the final dose of virus.

For assessing the effect of radiation and virus on tumour growth, HCT116 (2 × 10^6^ cells, n = 8), were inoculated subcutaneously on the right flank of athymic nude mice. Once a palpable tumour was apparent, tumour growth was monitored until a volume of 50 mm^3^ (V = LxWxDxpi/6) was reached, at which point animals were randomised to receive three doses of 0.5 × 10^10^ particles of EnAd-SA-FLuc virus in 50 µL PBS (n = 4) or 50 μL control PBS (n = 4), given i.v. 48 h apart. All animals received 10 Gy of irradiation to tumour 24 h after final dose. Tumours were monitored until a volume of 500 mm^3^ was reached.

### 4.11. Statistical Analysis

When comparing two data sets, a Student’s two-tailed t-test was used to analyse parametric data and a Mann–Whitney test to analyse non-parametric data. Where more than 2 groups were being compared, an analysis of variance (ANOVA) test was used with Bonferroni post hoc analysis to analyse parametric data and Kruskal–Wallis testing to analyse non-parametric data. All data is presented alongside bars indicating ± standard deviation (SD). The significant levels used were *p* = 0.01–0.05 (*), 0.001–0.01 (**), ≤0.001 (***).

## 5. Conclusions

The combination of radiotherapy and oncolytic virotherapy has the potential to revolutionize radiotherapy regimens and improve the efficacy of oncolytic virotherapies currently in clinical trials. Irradiation of target cells specifically increases EnAd particle release by upregulating adenovirus early gene expression. This increase in particles translates into improved spread and cytotoxicity, likely due to a loss in the cell’s ability to undergo DNA damage repair. Real-time monitoring of cytotoxicity using xCELLigence confirmed that there was a synergistic effect of radiotherapy and oncolytic virotherapy on target cell death in A549 cells in monolayers. An in vivo experiment was consistent with significant tumour growth inhibition of HCT116 xenografts when three doses of EnAd were given prior to a single 10 Gy dose of radiation, suggestive of a radiosensiting effect in this tumour model. Our results will form the framework for further molecular studies to complement the currently recruiting CEDAR trial.

## Supplementary Materials

The following are available online at https://www.mdpi.com/2072-6694/12/4/798/s1, Figure S1: CD46 and DSG2 expression in infected cells after irradiation, Figure S2: 53BP1 and Rad51 foci formation in infected and irradiated cells, Figure S3: EnAd infection is associated with phosphorylation of 53BP1 in the absence of irradiation in HCT116 cells, Figure S4: EnAd E1A expression is enhanced in pre-irradiated cells, Figure S5: EnAd modulates the DNA damage response in infected cells, Figure S6: Enadenotucirev infection is associated with a partially proteasomally mediated decrease in DNA ligase IV levels, Figure S7: Ad5 E1A expression is enhanced in pre-irradiated HCT116 but not A549 cells, Figure S8: EnAd infection is associated with phosphorylation of 53BP1 in the absence of irradiation in HCT116 cells, shown here are uncropped blots seen in Figure S3.
