# Supplementary material for: External Beam Radiation Therapy and Enadenotucirev: Inhibition of the DDR and Mechanisms of Radiation-Mediated Virus Increase
Source: Cancers (Basel). 2020 Mar 26;12(4):798. doi: 10.3390/cancers12040798 (PMC7226394; doi:10.3390/cancers12040798)
Supplement: Supplementary file 1 [file cancers-12-00798-s001.pdf]

Supplementary Materials: External Beam Radiation Therapy and Enadenotucirev: Inhibition of the DDR and Mechanisms of Radiation-Mediated Virus Increase

Tzveta D. Pokrovska, Egon J. Jacobus, Rathi Puliyadi, Remko Prevo, Sally Frost, Arthur Dyer, Richard Baugh, Gonzalo Rodriguez-Berriguete, Kerry Fisher, Giovanna Granata, Katharine Herbert, William K. Taverner, Brian R. Champion, Geoff S. Higgins, Len W. Seymour and Janet Lei-Rossmann

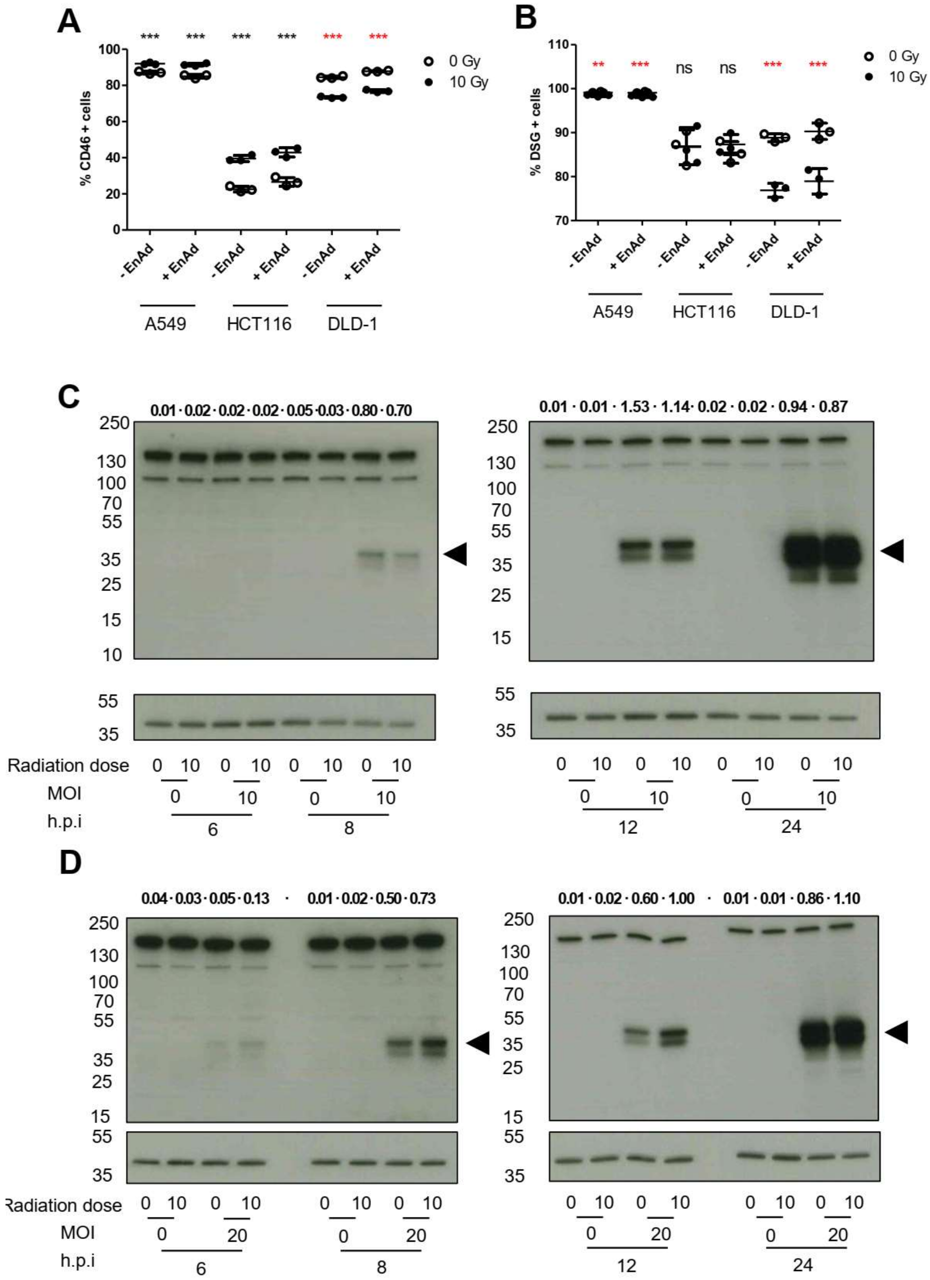

Figure S1. CD46 and DSG2 expression in infected cells after irradiation. A549 cells were seeded into 12-well plates. A549, HCT116, or DLD-1 cells were mock-infected or infected with EnAd-SA-GFP the next day at an MOI of 0.1. At 24 h p. i. cells were mock-irradiated or irradiated at 10 Gy. Supernatant and cells were harvested 48 h p. i. Percentage of positive cells are shown for CD46 (**A**) and DSG2 (**B**). Figure shows mean ± SD of a single experiment

representative of three experimental repeats. Each point represents data for a single well. Significance was evaluated using 2-way ANOVA with Bonferroni post-test. $p < 0.05$: *, $p < 0.01$: **, $p < 0.001$: ***. (**C**) A549 or (**D**) HCT116 cells were irradiated at 10 Gy and seeded after eight hours. Cells were infected with Ad5 24 h post-irradiation at an MOI of 0, 10 (**C**) or 0, 20 (**D**). At six, eight, twelve or twenty four hours post-infection, 30 µg of cell lysates were harvested and probed using an anti-E1A antibody or β-actin (loading control). Density for each band is given above the corresponding lane as a β-actin-corrected value.

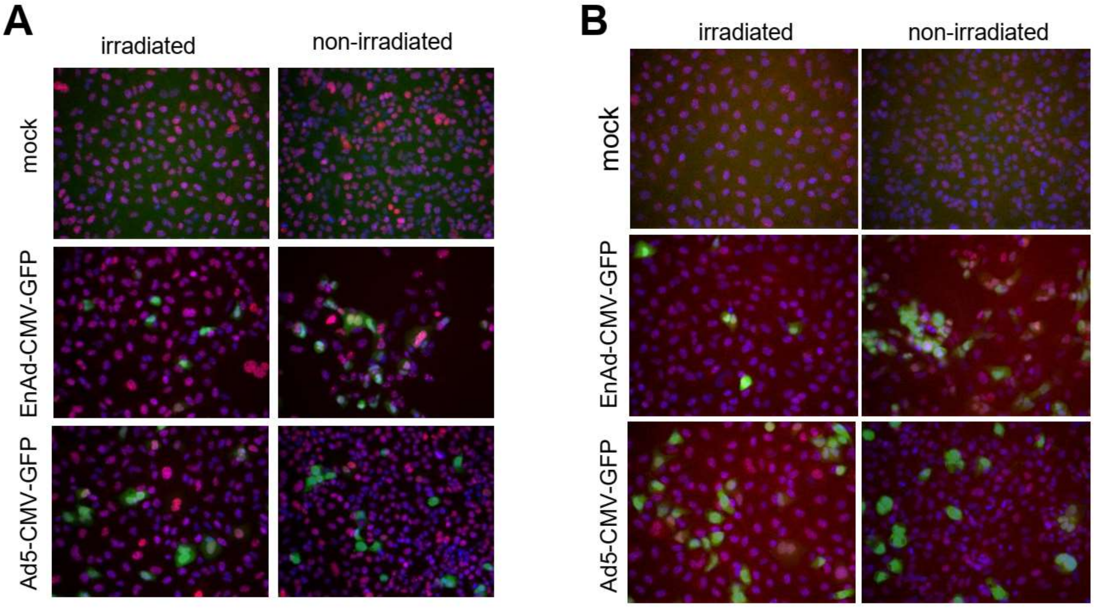

Figure S2. 53BP1 and Rad51 foci formation in infected and irradiated cells. A549 cells were mock-infected or infected with EnAd-CMV-GFP or Ad5-CMV-GFP at 500 VP/cell. At 24 h post-infection (p. i.), cells were either irradiated at 6 Gy or left non-irradiated. Cells were fixed at different time points following irradiation and subsequently blocked and stained for DAPI and 53BP1 (**A**) or Rad51 (**B**). Images show representative staining patterns in cells 2 h (**A**), 6 h (**B**), or 24 h post-irradiation in mock-irradiated plates.

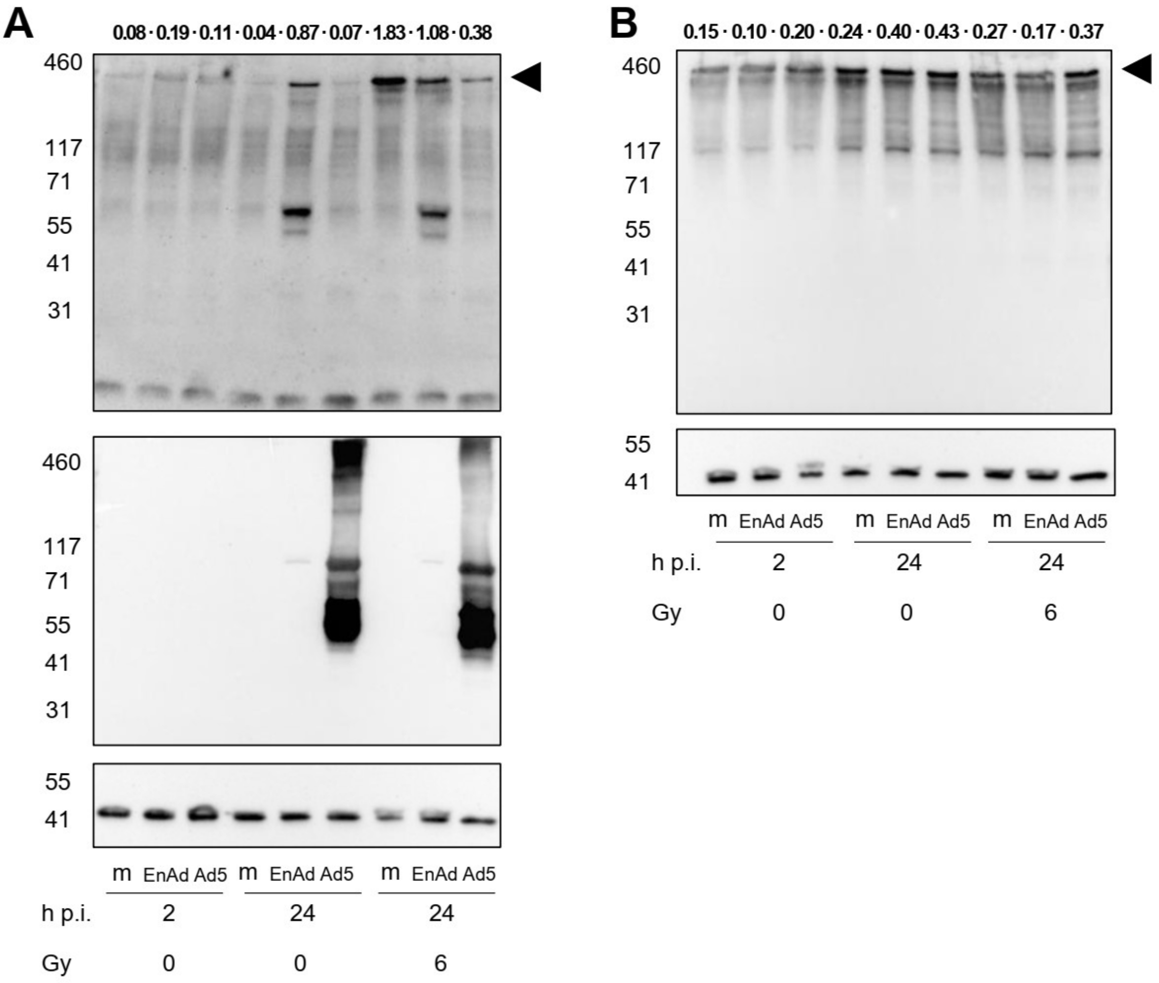

Figure S3. EnAd infection is associated with phosphorylation of 53BP1 in the absence of irradiation in HCT116 cells. HCT116 cells were seeded into 6-well plates and the next day mock-infected or infected with EnAd-SA-GFP or Ad5 at an MOI of 100. Cell lysate was harvested 2 h p.i. and 24 h p.i. into RIPA buffer. Two hours prior to the 24 h time point, half of the samples were irradiated at 6 Gy and the other half mock-irradiated. Each lane contains 50 μg of protein. (**A**) Top panel shows results for antibody to 53BP1 phosphorylated at serine 1778. Middle panel shows results for polyclonal antibody to late adenovirus structural proteins. Bottom panel shows staining for β-actin. (**B**) Top panel shows staining for antibody to total 53BP1 and bottom panel shows staining for β-actin. m, mock; h p.i., hours post-infection; Gy, radiation dose given in Gray. Density for each band is given above the corresponding lane as a β-actin-corrected value.

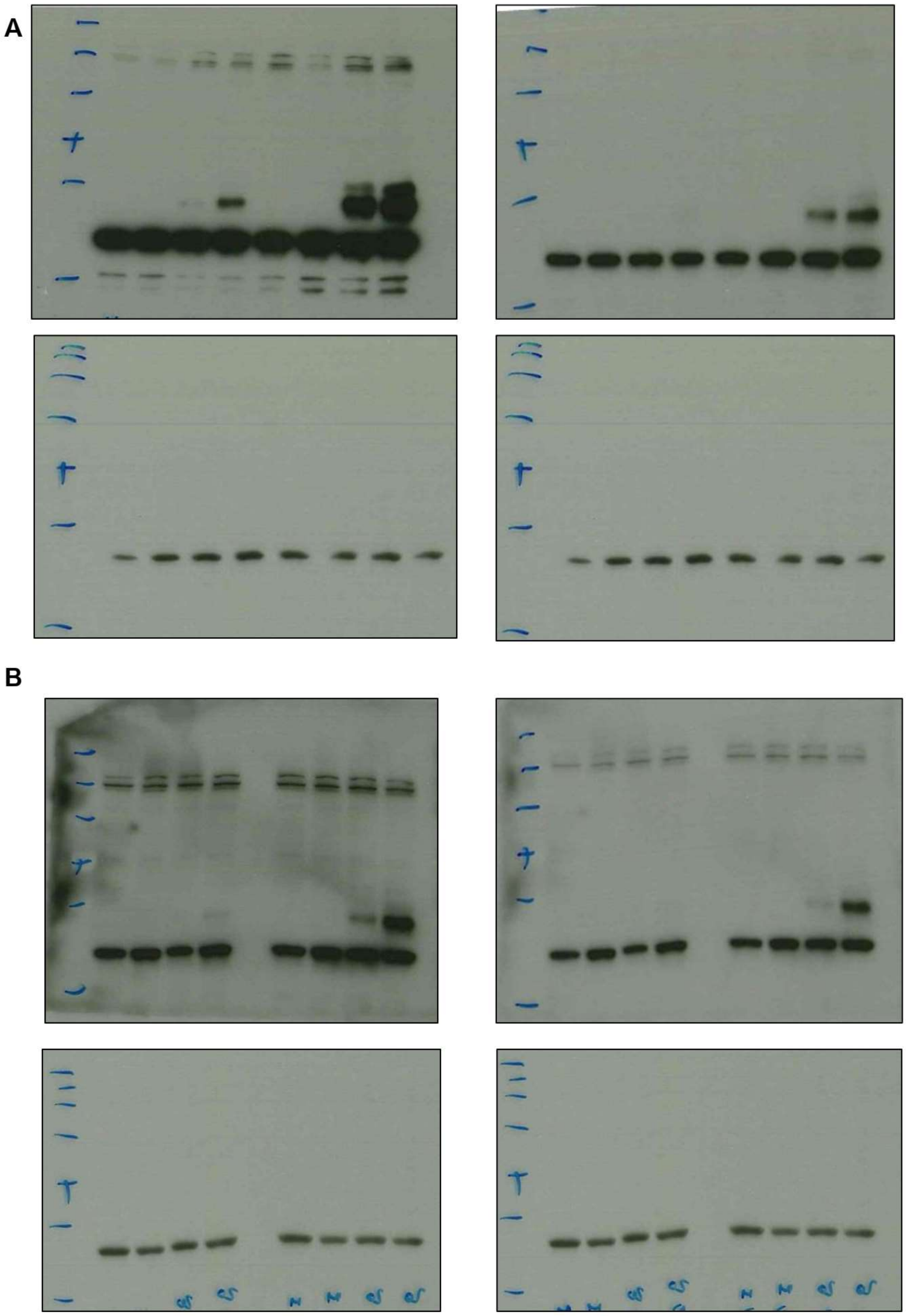

Figure S4. EnAd E1A expression is enhanced in pre-irradiated cells. (**A**) A549 or (**B**) HCT116 cells were irradiated at 10 Gy and seeded after eight hours. Cells were infected with EnAd-E1a-FLAGtag 24 h post-irradiation at an MOI of 0 or 10 (**A**) or an MOI 0 of 20 (**B**). At eight hours and twelve hours post-infection, 70 μg of cell lysates were harvested and probed using an anti-FLAG antibody or β-actin (loading control). Above blots show whole uncropped blots seen in Figure 2A,B, respectively.

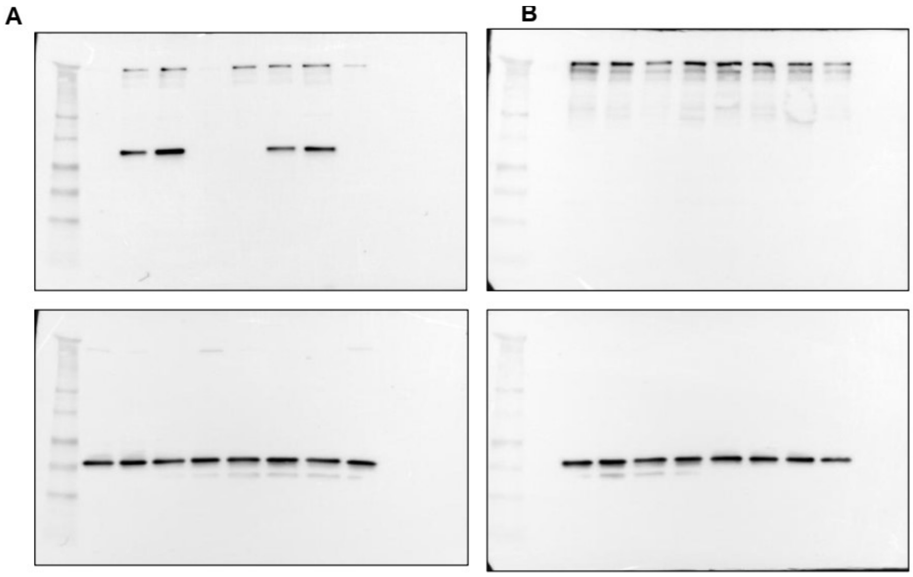

Figure S5. EnAd modulates the DNA damage response in infected cells. (**A**,**B**) A549 cells were mock-infected or infected with EnAd-SA-GFP, Ad11p or Ad5 at an MOI of 100. Cells were irradiated 22 h p. i. and harvested 24 h p. i. Cell lysates (20 µg/lane) were separated by SDS-PAGE and probed using an antibody against Ser1778- phosphorylated 53BP1 (**A**), total 53BP1 (**B**) or β-actin. Western blots above are uncropped blots seen in Figure 3D,E, respectively.

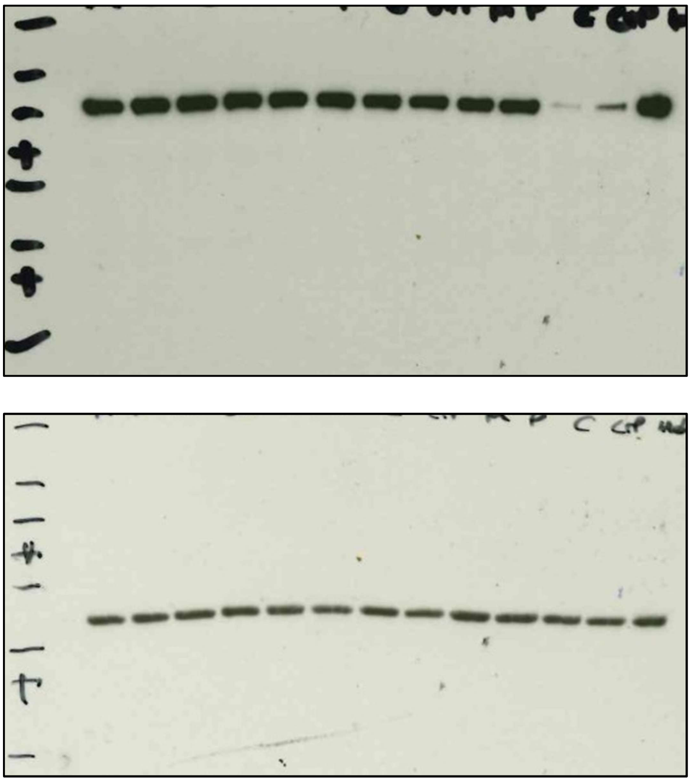

Figure S6. Enadenovirus infection is associated with a partially proteasomally-mediated decrease in DNA ligase IV levels. A549 cells were infected with EnAd SA-GFP at 200 VP/cell (approximately an MOI of 11) or mock-infected. At 2 h post-infection, cells were treated with 10 µM MG132 or DMSO control. Cell lysates were harvested either immediately after treatment, 24 h p. i., or 48 h p. i., separated by SDS-PAGE and probed with antibodies against DNA ligase IV or β-actin. Lanes contain 30 µg of protein. Data is representative of three repeats. Figure shows uncropped blots seen in Figure 4.

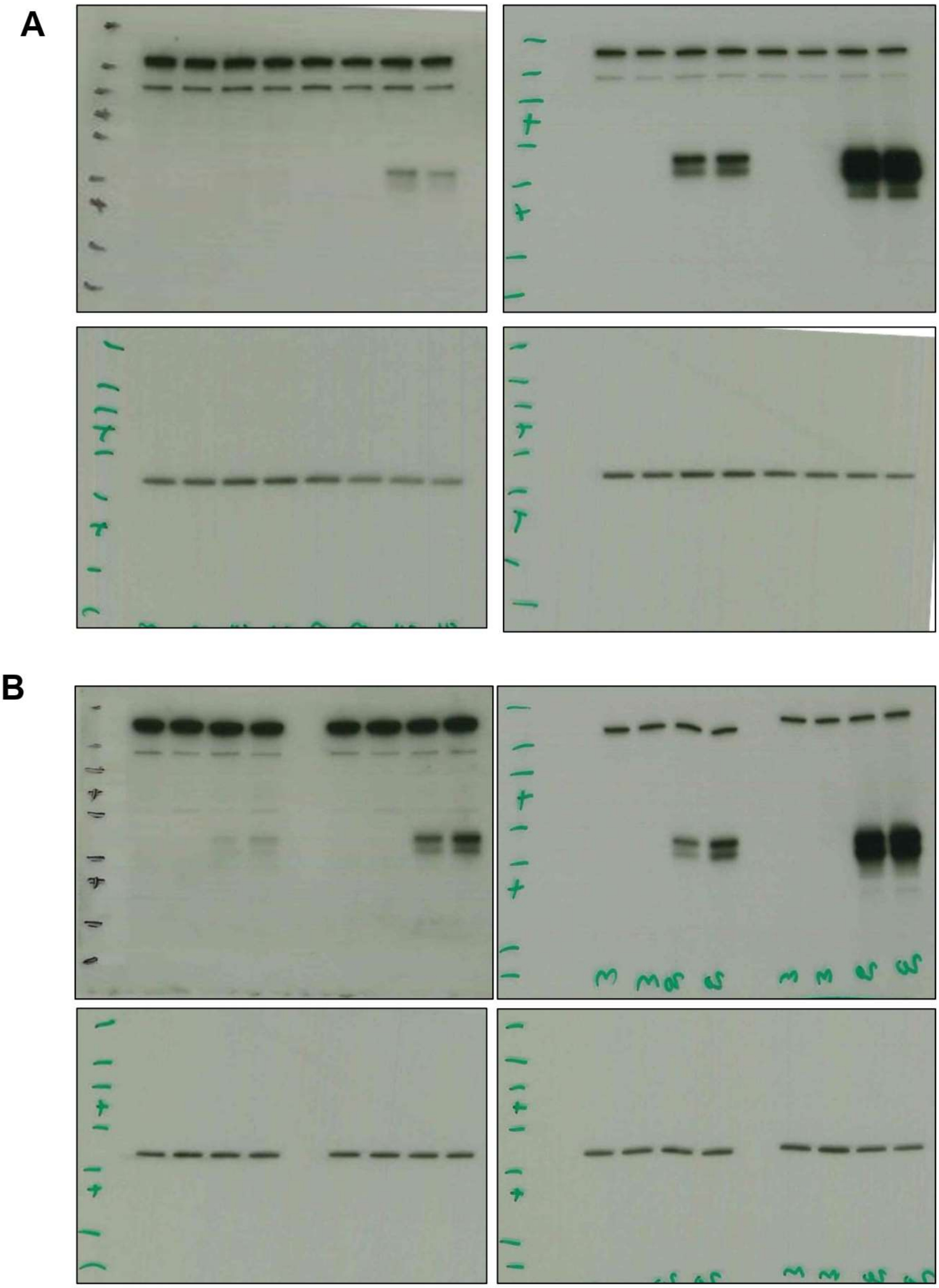

Figure S7. Ad5 E1A expression is enhanced in pre-irradiated HCT116 but not A549 cells. (**A**) A549 or (**B**) HCT116 cells were irradiated at 10 Gy and seeded after eight hours. Cells were infected with Ad5 24 h post-irradiation at an MOI of 0, 10 (**A**) or 0, 20 (**B**). At six, eight, twelve or twenty four hours post-infection, 30 μg of cell lysates were harvested and probed using an anti-E1A antibody or β-actin (loading control). Blots show uncropped blots from supplementary Figure 1C,D, respectively.

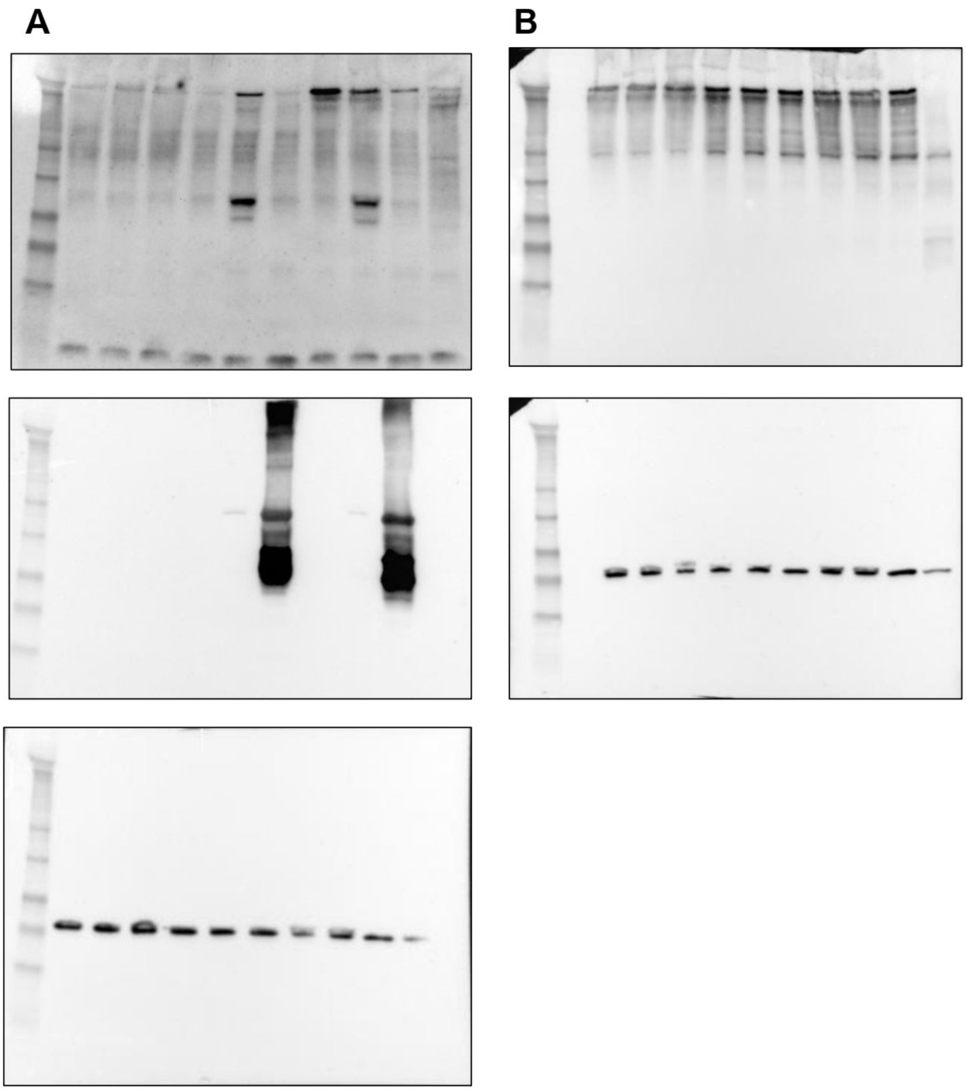

Figure S8. EnAd infection is associated with phosphorylation of 53BP1 in the absence of irradiation in HCT116 cells. HCT116 cells were seeded into 6-well plates and the next day mock-infected or infected with EnAd-SA-GFP or Ad5 at an MOI of 100. Cell lysate was harvested 2 h p.i. and 24 h p.i. into RIPA buffer. Two hours prior to the 24 h time point, half of the samples were irradiated at 6 Gy and the other half mock-irradiated. Each lane contains 50 μg of protein. (**A**) Top panel shows results for antibody to 53BP1 phosphorylated at serine 1778. Middle panel shows results for polyclonal antibody to late adenovirus structural proteins. Bottom panel shows staining for β-actin. (**B**) Top panel shows staining for antibody to total 53BP1 and bottom panel shows staining for β-actin. Shown here are uncropped blots seen in Figure S3.

© 2020 by the authors. Licensee MDPI, Basel, Switzerland. This article is an open access article distributed under the terms and conditions of the Creative Commons Attribution (CC BY) license (http://creativecommons.org/licenses/by/4.0/).
